# Case report of traumatic intrapericardial diaphragmatic hernia: Laparoscopic composite mesh repair and literature review

**DOI:** 10.1016/j.ijscr.2020.04.077

**Published:** 2020-05-07

**Authors:** Emad A. Aborajooh, Zaid Al-Hamid

**Affiliations:** a Department of General Surgery, Mutah University, Kerak, 61710, Jordan; b Department of General Surgery, Blackpool Teaching Hospital NHS Foundation Trust, UK

**Keywords:** Intrapericardial diaphragmatic hernia, Diaphragmatic hernia, Composite mesh, Hernia

## Abstract

**Introduction:**

Traumatic rupture of the diaphragm with herniation of abdominal viscera into the pericardium is a rare injury. We present a case of intrapericardial diaphragmatic hernia after one year of thoracoabdominal trauma. This case is the 106th in the literature and only the fifth treated by the laparoscopic approach including this case. After case presentation, we will discuss post-traumatic intrapericardial diaphragmatic hernias by reviewing the literature.

**Case presentation:**

A 48-year-old male presented to our outpatient clinic with a history of thoracoabdominal trauma due to a motor vehicle accident one year ago. The patient complained from abdominal pain and constipation over the last year. After a thorough history and physical examination, thoracoabdominal computed tomography (CT) was performed. The CT scan revealed herniation of the transverse colon and omentum through a large anterior diaphragmatic defect into the pericardial sac. Laparoscopic composite mesh repair was done. The patient had an uneventful postoperative course and was discharged home on the second postoperative day. The patient was asymptomatic at regular follow-up six months postoperatively.

**Conclusion:**

Late presentation of intrapericardial diaphragmatic hernia is rare. Chest computed tomography is the most useful diagnostic tool. Delayed diagnosis of Intrapericardial diaphragmatic hernia does not preclude laparoscopic repair by primary closure or with mesh according to the size of the defect.

## Highlights

• Post-traumatic rupture of the diaphragm with herniation of abdominal viscera into the pericardium is a rare injury.
• Delayed diagnosis of Intrapericardial diaphragmatic hernia does not preclude laparoscopic repair.
• Repair of large defect with composite mesh reduces the risk of adhesions, infection and erosion of nearby viscera.

## 1 Introduction

The diaphragm is an important musculoaponeurotic structure that separates the thoracic and abdominal cavities. The diaphragm is originated between fourth to twelfth weeks of gestation from septum transversum, pleuroperitoneal membranes, mediastinal dorsal mesentery of the esophagus and the body wall muscles [1]. The diaphragm is formed by three muscular parts: costal, crural and minor sternal diaphragm. The insertion of these three muscular parts is a thickened fascial aponeurosis called central tendon. The pericardium is located above the central part of the tendon and attached to its superior surface [2].

Diaphragmatic hernia caused by congenital defects or trauma (blunt or penetrating). The estimated prevalence of traumatic diaphragmatic hernia is 3–7% of all abdominal and thoracic traumas [3]. Herniation of abdominal viscera into the pericardium is very rare [3]. In this case report, we present a case of delayed post-traumatic intrapericardial diaphragmatic hernias and the current literature is reviewed. Up to the best of our knowledge, there are 106 cases in the literature and only five cases treated by the laparoscopic approach (including this one). The case has been reported in line with the SCARE criteria [4].

## 2 Case report

**Fig. 1:**
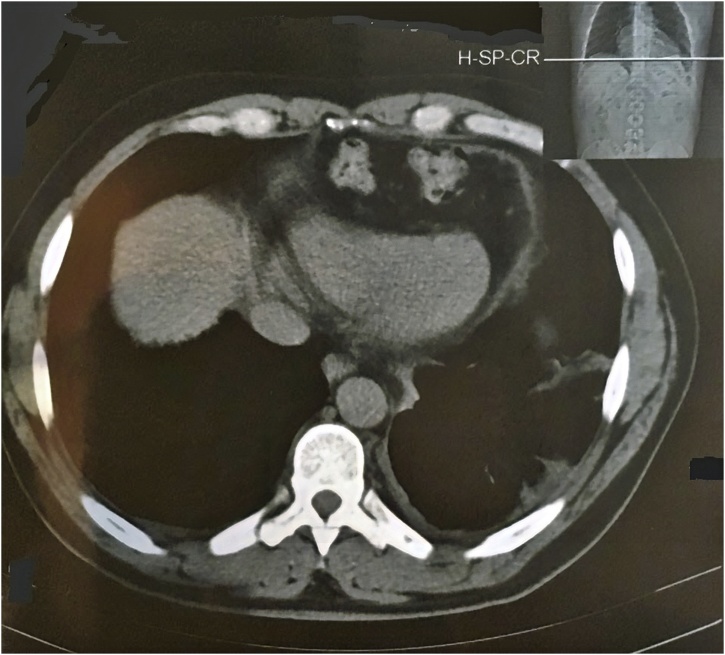
Computed Tomography (CT) scan revealed herniation of the transverse colon and omentum through a large anterior diaphragmatic defect into the pericardial sac.

**Fig. 2:**
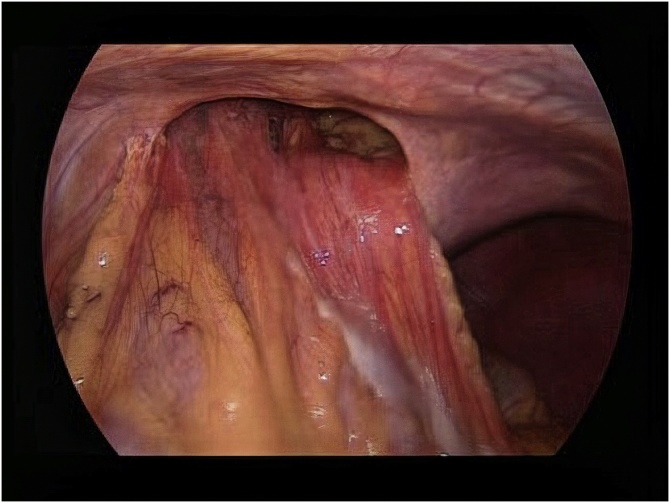
A defect in the central area of the diaphragm measured 12 × 7 cm with greater omentum and part of transverse colon herniated into the pericardial sac.

**Fig. 3:**
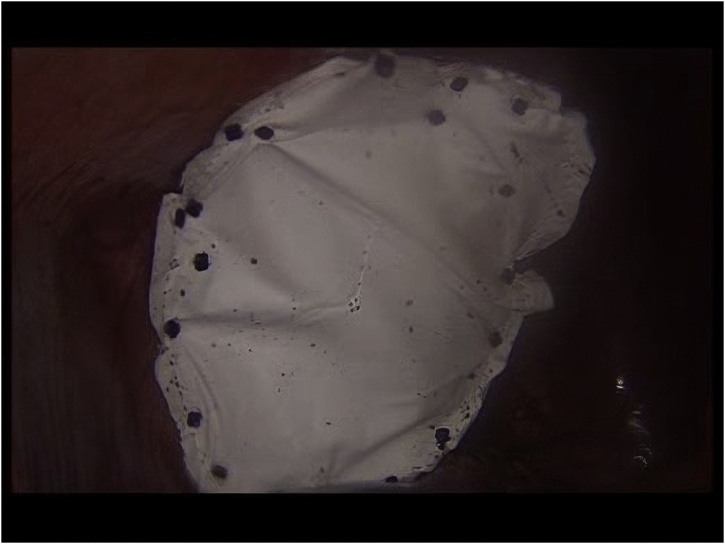
Composite mesh in situ secured with absorbable tacks to close the large diaphragmatic defect.

A 48-year-old male presented to our outpatient clinic with a history of thoracoabdominal trauma due to a motor vehicle accident one year ago. The patient was complaining of abdominal pain and constipation over the last year. After a thorough history and physical examination, thoracoabdominal computed tomography (CT) was performed. The CT scan revealed herniation of the transverse colon and omentum through a large anterior diaphragmatic defect into the pericardial sac (Fig. 1). Laparoscopic surgery was done with a 10 mm camera port 3 cm above the umbilicus and another 2 working ports at the left (5 mm) and right (12 mm) midclavicular subcostal margin. Pneumoperitoneum by CO_2_ gas was maintained at 12 cm H_2_O and with reverse Trendelenburg modified lithotomy position. A defect measured 12 × 7 cm was seen in the central area of the diaphragm. Greater omentum and part of transverse colon herniated into the pericardial sac (Fig. 2). Adhesiolysis and reduction of abdominal content were done using non-traumatic intestinal forceps and LigaSure™ device. The diaphragmatic defect was repaired by composite mesh as primary closure precluded by large defect size. The mesh was oriented and secured in a tension free manner with absorbable tacks (Fig. 3). No drain was placed and the patient had an uneventful postoperative course. The patient was discharged home on the second postoperative day. The patient was asymptomatic at regular follow-up six months postoperatively.

## 3 Literature review

Keith reported the first two cases of traumatic intrapericardial diaphragmatic hernia (TIPDH) as autopsy findings in 1910 [1]. In 1986, Van Lohenhout et al. reviewed the literature and reported 58 cases of TIPDH and reported a new case [5]. Reina et al. reviewed the literature from 1986–1999 and reported a total of 23 cases including their case [3]. Another two cases of TIPDH during this period between 1986–1999 were reported by Rodriguez-Morales et al. [6]. Kuy et al. reviewed the literature from 1999 to 2012 and found another 11 cases [7].

By reviewing the literature, four additional cases of TIPDH were found before 2012 that were not mentioned by Kuy et al. Since 2012, five additional cases of TIPDH were reported in the literature. This review includes 10 cases of TIPDH including the presented case, so the total number of cases became 106 cases.

Our patients was treated with laparoscopic approach and it is the fifth reported in literature and the second to be repaired with composite mesh. Table 1 shows the demographic data, mechanism of injury, timing of diagnosis, presenting symptoms, imaging modality, herniated viscera, defect size, surgical approach and type of repair of the 10 cases. Upon review of the current literature, the age of patients ranges from 43 to 78 years with mean age 56.3 years. We observed male predominance in the reported cases. The most common cause was blunt trauma due to motor vehicle collision. Acute presentation of chest pain, shortness of breath and abdominal pain were observed in four cases. Other cases of TIPDH presented with chronic or recurrent vague symptoms of dyspepsia, abdominal pain, malaise and shortness of breath accompanied by the history of previous remote trauma ranging from one year to 23 years before the diagnosis. The most yielding imaging modality was computed tomography. The diameter of defect ranged between 3.5 cm and 10 cm. The encountered organ inside the hernia were omentum, stomach, transverse colon, small bowel and left lobe of the liver in descending order. Laparotomy was the most commonly performed surgical approach with other incisions such as thoracotomy, thoracoscopy and median sternotomy were utilized according to the case. In 2009, Al-Ghnaniem et al. described the first successful primary repair of intrapericardial diaphragmatic hernia using a laparoscopic approach [8]. Kuy et al. also utilized laparoscopy for primary repair of large diaphragmatic defects [7]. The first laparoscopic mesh repair was described by McCutcheon et al. in 2010 [9]. Four years later another case of laparoscopic mesh repair reported by Burneo Esteves et al. [10]. Finally, we describe a laparoscopic composite mesh repair for a diaphragmatic defect measuring 12 × 7 cm. There is no documented immediate or late postoperative complication in all five cases treated by laparoscopic approach including the presented case that showed uneventful postoperative course.

**Table 1:** Summary of ten cases of post-traumatic intrapericardial diaphragmatic hernia.

Author, year	Age (years)	Gender	Mechanism	Time to Diagnosis	Presenting symptoms	Imaging	Herniated organs	Defect Size	Surgical Approach/Type of repair
Castedo et al., 2004 [14]	66	Male	MVC^‡^	Acute	Chest and pelvic pain	Chest roentgenogram	Stomach	NA^¥^	Laparotomy/primary repair
Nejmeddine et al., 2007 [15]	56	Male	MVC^‡^	Acute	Abdominal pain and hypotesion	CT	Stomach and omentum	10 cm	Laparotomy/primary repair
Al-Ghnaniem et al., 2009 [8]	46	Male	Stabbing	Acute	Chest pain, abdominal pain and distension	CT	Omentum	4.5 cm	Laparoscopy/primary repair
Choi et al., 2015 [16]	43	Male	MVC^‡^	Acute	Orthopnea and epigastric pain	CT	Stomach and omentum	8 cm	Thoracoscopy, and laparotomy/primary repair
Nwafor et al., 2011 [17]	49	NA^¥^	MVC^‡^	2 years	Fatigability and palpitations	CT	Stomach and coils of small bowel	NA^¥^	Thoracotomy/primary repair
Cipe et al., 2012 [18]	NA^¥^	NA^¥^	MVC^‡^	7 years	Postprandial abdominal pain and dyspnea	CT and CXR	Stomach, transverse colon and omentum	large (no dimensions)	Laparotomy/NA^¥^
Burneo Esteves et al. 2014 [10]	NA^¥^	NA^¥^	Trauma	NA¥	Dyspepsia and epigastric pain	CT	Transverse colon and omentum	10 × 7 cm	Laparoscopy/repair with mesh
Jagana et al. 2014 [19]	NA^¥^	NA^¥^	Trauma	23 years	Abdominal pain, nausea,	CT	Loop of small bowel	NA^¥^	Laparotomy/NA^¥^
Spiliotopoulos et al. 2017 [20]	78	Female	MVC^‡^	13 years	Cough, dyspnea and malaise	CT angiogram	Left lobe of the liver, fat mesentery and bowel loops(large and small)	diameter, 3.6 cm within the neck	Median sternotomy- pericardial patch and Prolene sutures
current case 2020	48	Male	MVC^‡^	1 year	Abdominal pain and constipation	CT	Transverse colon and omentum	12 × 7 cm	Laparoscopy-repair with mesh

MVC ‡: motor vehicle collision; NA ¥: Not Available.

## 4 Discussion

The traumatic diaphragmatic hernias mostly occur at anatomic weakness where the diaphragm attaches to the chest wall in the left posterolateral aspect. The blunt abdominal trauma may rarely cause central tendon rupture and give the access of the abdominal viscera to herniate into the pericardial sac. As the pericardial space has limited space, abdominal viscera herniation into it mandates surgical repair to prevent life-threatening cardiac tamponade [3].

The surgical approach for TIPDH repair is not clear yet. Nevertheless, laparotomy is the preferred approach for acute presentation and thoracotomy for chronic delayed presentation owing to more feasible for adhesiolysis. Other authors reported that the laparoscopic, either primarily or with mesh, approach is a safe, feasible and effective for both acute and chronic TIPDH repair. Some reports showed that there were no significant adhesions between the heart itself and the abdominal viscera. Adhesions between the abdominal viscera and the pericardial lining can be easily divided using harmonic scalpel, and this is what we experienced in the this case [9,11]. Acute traumatic diaphragmatic defects can be repaired primarily as the diaphragmatic defect edges are pliable and tractable. On the contrary, delayed presentation of the diaphragmatic defect and large defects preclude primary closure as the edges become weak, thin, non-tractable and might not hold sutures [12]. In such cases, closure of the defect by synthetic mesh is appealing and provide robust support. Despite its wide availability, durability and cost-effectiveness, synthetic mesh is associated with many complications as infection, erosion into surrounding viscera, and adhesions [13]. This raises the need to try other types of prosthesis as composite and biological mesh despite its high cost. There are clear advantages of composite mesh use in ventral abdominal hernias but such advantages cannot be concluded in the diaphragmatic defect repair due to limited reports in the literature [12]. In this case, primary closure was not feasible as the diaphragmatic edges could not hold the suture and the diaphragmatic defect was large. Composite mesh was used to maintain the diaphragmatic optimum function during heart beating and to reduce the risk of adhesions, infection and erosion of nearby viscera that can be caused by conventional synthetic mesh.

## 5 Conclusion

Delayed intrapericardial diaphragmatic hernia is a rare presentation after trauma. Chest computed tomography is the most diagnostic tool of choice. Delayed diagnosis of Intrapericardial diaphragmatic hernia does not preclude laparoscopic repair by either primary closure or with mesh for large defects.

## Declaration of Competing Interest

No conflict of interest.

## Sources of funding

This research did not receive any specific grant from funding agencies in the public, commercial, or not-for-profit sectors.

## Ethical approval

Ethical approval for this study was obtained from Mu’tah faculty of Medicine Ethics committee (Reference number. 202029).

Based on our ethical considerations and guidelines, the committee has approved the project without amendments.

Statement in the publication”Ethical approval was obtained from Mu’tah faculty of Medicine Ethics committee”.

## Consent

Written informed consent was obtained from the patient for publication of this case report and accompanying images. A copy of the written consent is available for review by the Editor-in-Chief of this journal on request.

## Author contribution

**Aborajooh A. Emad**: study concept or design, data collection, data analysis or interpretation, writing the paper.

**Al-Hamid Zaid**: study concept or design, data collection, data analysis or interpretation, writing the paper.

## Registration of research studies

1. Name of the registry: Traumatic Intrapericardial Diaphragmatic Hernia: Laparoscopic Composite Mesh Repair and Literature Review.
2. Unique Identifying number or registration ID: researchregistry5384.
3. Hyperlink to your specific registration (must be publicly accessible and will be checked). https://www.researchregistry.com/browse-the-registry#home/.

## Guarantor

Aborajooh A. Emad.

Department of General Surgery, Mutah University, Al-Karak, 61710, Jordan.

## Provenance and peer review

Editorially reviewed, not externally peer-reviewed.
